# Pancreatoduodenectomy for Gastric Cancer With Pancreatic Involvement: Indications, Outcomes, and Prognostic Factors

**DOI:** 10.7759/cureus.101563

**Published:** 2026-01-14

**Authors:** Sergio Isidro Gamboa-Hoil

**Affiliations:** 1 Surgical Oncology, Mexican Social Security Institute, Merida, MEX

**Keywords:** gastric cancer, pancreatic involvement, pancreatoduodenectomy, patient selection, prognostic factors, r0 resection, surgical outcomes

## Abstract

Pancreatoduodenectomy (PD) is rarely indicated for gastric cancer because of its high morbidity and uncertain survival benefit; however, in selected patients with gastric cancer and pancreatic involvement, PD may be required to achieve complete oncologic resection. This narrative review summarizes current evidence regarding indications, outcomes, and prognostic factors associated with PD in gastric cancer with pancreatic invasion. A narrative review of the literature was conducted focusing on published series evaluating clinicopathologic features, surgical management, perioperative outcomes, survival, and prognostic factors in patients undergoing PD for gastric cancer with pancreatic involvement. Pancreatic involvement is an uncommon finding in gastric cancer; however, PD is considered only in carefully selected patients when direct pancreatic invasion precludes margin-negative resection with standard gastrectomy. Tumors are most frequently located in the distal stomach and gastric antrum and are typically associated with advanced local disease. Preoperative imaging and intraoperative assessment have limited accuracy in distinguishing true pancreatic invasion from inflammatory adhesions, making histopathologic confirmation essential. Across published series, R0 resection can be achieved in a substantial proportion of patients; however, postoperative morbidity remains considerable, with pancreatic fistula being the most commonly reported complication. Long-term survival outcomes are highly variable and appear to be primarily influenced by tumor biology and the achievement of margin-negative resection. Consistently, the achievement of R0 resection emerges as the most important favorable prognostic factor. PD should not be routinely performed for gastric cancer but may be justified in carefully selected patients with pancreatic invasion when required to achieve complete oncologic resection, with outcomes driven more by oncologic factors than by the extent of surgery itself.

## Introduction and background

The pancreas is one of the most frequently involved adjacent organs in advanced gastric cancer, with reported rates of pancreatic involvement reaching up to 52.1% in locally advanced cases, and it represents the most commonly resected organ during multivisceral resections, accounting for approximately 41.1% of such procedures [1-4]. In gastric cancer surgery, achieving complete tumor removal remains a fundamental oncologic principle, as margin-negative (R0) resection has consistently been identified as the most important determinant of long-term survival across multiple studies [3,5,6].

Despite this oncologic rationale, pancreatoduodenectomy (PD) for gastric cancer has historically been considered highly controversial because of its technical complexity, substantial perioperative morbidity, and uncertain survival benefit [3,4,7]. Early experiences with PD in this setting were discouraging. In 1978, Buchholtz et al. reported poor outcomes and concluded that PD should not be performed for gastric cancer due to an unacceptable operative risk [8].

However, advances in surgical technique, perioperative management, critical care, and patient selection have led to renewed interest in PD for carefully selected patients with gastric cancer and suspected pancreatic invasion [3,4]. In recent years, PD has been explored in highly selected patients as a strategy to achieve R0 resection when standard gastrectomy is insufficient [3,6]. Nevertheless, reported outcomes remain heterogeneous, and the true oncologic benefit of PD continues to be debated [2,3].

This clinical scenario does not readily lend itself to prospective randomized or double-blind clinical trials, given its rarity, biological heterogeneity, and the frequent need for intraoperative decision-making to distinguish true pancreatic invasion from inflammatory adherence and to assess en bloc tumor resectability. Consequently, available evidence largely derives from retrospective series and high-volume institutional experiences [3].

In this context, the present narrative review aims to synthesize the available evidence regarding the indications, perioperative outcomes, survival, and prognostic factors associated with PD in patients with gastric cancer and pancreatic involvement, with the goal of clarifying its potential role in contemporary gastric cancer surgery and informing surgical decision-making.

## Review

Search strategy and methods

A non-systematic literature search was conducted using PubMed, MEDLINE, and Google Scholar to identify relevant studies published between 1999 and 2024 evaluating PD for gastric cancer with pancreatic involvement. Search terms included combinations of “gastric cancer”, “pancreatic invasion”, “pancreatic involvement”, “pancreatoduodenectomy”, “extended resection”, “R0 resection”, and “prognostic factors”.

The search focused on English-language publications reporting clinicopathologic characteristics, surgical indications, perioperative outcomes, survival, and prognostic factors in patients undergoing PD for gastric cancer invading the pancreas. Reference lists of key articles were manually reviewed to identify additional relevant studies.

Given the rarity of this clinical scenario, the available evidence consisted primarily of retrospective series and observational studies. All study designs providing original clinical data were considered eligible for inclusion.

Inclusion and exclusion criteria

Studies were included if they were published in English and reported outcomes of patients with gastric cancer and pancreatic involvement undergoing PD, with available data on surgical outcomes, survival, or prognostic factors. Studies were excluded if they consisted solely of case reports, did not specifically address pancreatic involvement, or lacked relevant oncologic or surgical outcome data.

Risk of bias consideration

Given the narrative nature of this review and the limited number of available studies, no formal risk-of-bias assessment tool was applied. However, the methodological limitations of the included studies, including small sample sizes and retrospective design, are acknowledged and discussed where relevant.

Incidence

Approximately 4.8% of all patients with gastric cancer present with pancreatic involvement [9], and between 11% and 39% of those with pancreatic invasion require PD [9,10]. Among patients undergoing PD, 78% had primary gastric cancer, whereas 22% underwent PD for carcinoma arising in the gastric remnant following a previous Billroth I gastrectomy [10].

The reported age at diagnosis ranges from 42 to 64 years, with overall age ranges between 31 and 81 years [4,10-13]. A male predominance has been described, with male-to-female ratios ranging from 1.2:1 to 3.6:1 [4,6,10,12].

Tumor location

**Figure 1:**
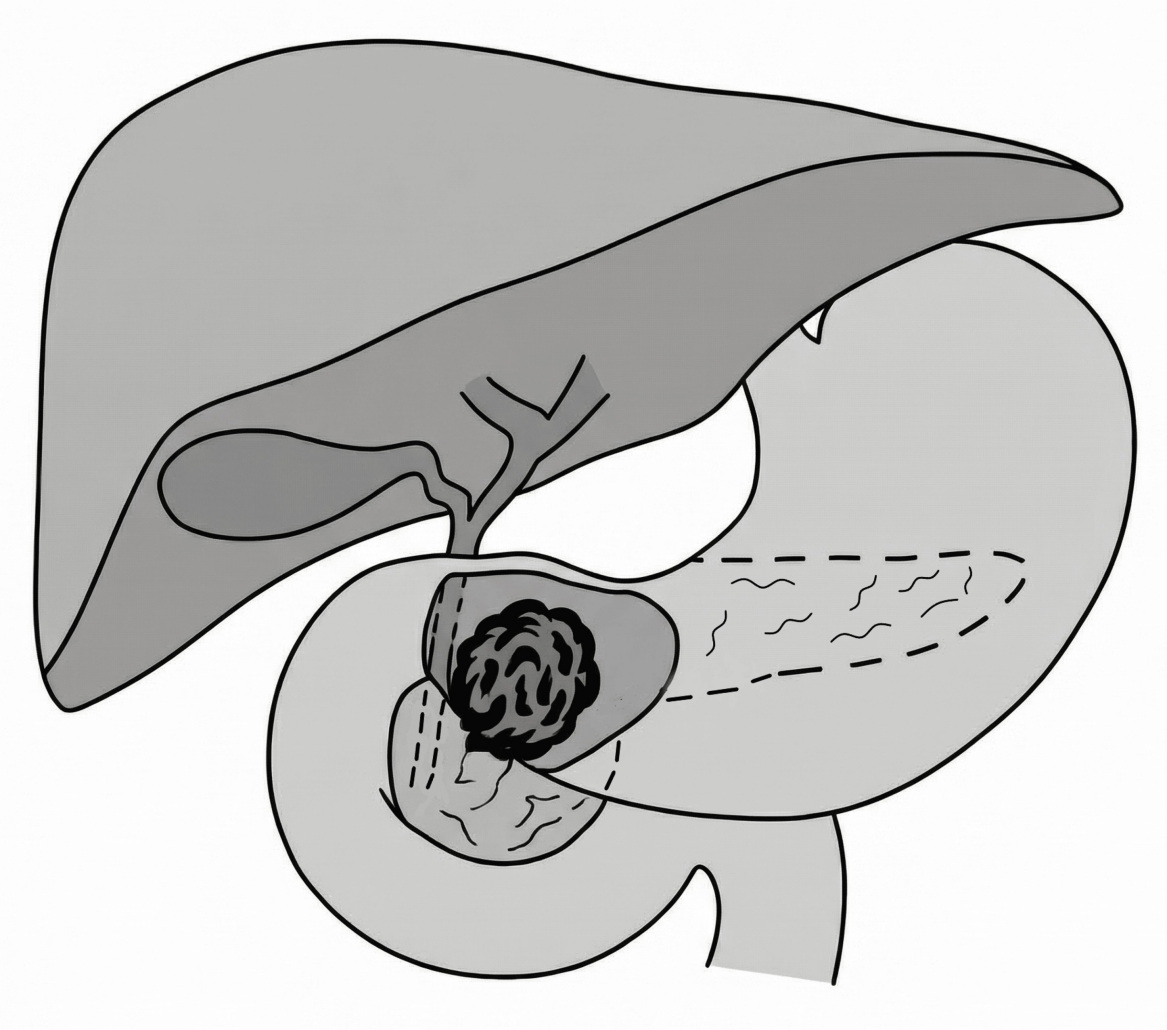
Schematic representation of distal gastric cancer with direct invasion of the pancreatic head. The illustration demonstrates the anatomic relationship between the distal stomach, duodenum, and pancreas, highlighting a locally advanced tumor extending into the pancreatic head, a scenario in which pancreatoduodenectomy may be required to achieve R0 resection. Figure created by the author.

In patients with gastric cancer, involvement of the distal third of the stomach has been reported in 34.7-54.3% of cases, with tumors larger than 5 cm observed in 65.4% [6,14,15]. These findings may help explain why, in series requiring PD, tumors predominantly involved the gastric antrum (78%) and frequently demonstrated extension into the duodenum (61%) (Figure 1) [10].

Symptoms

Clinical symptoms are present in 91.3% of patients, with abdominal pain reported in 56%. Obstructive symptoms, including early satiety and vomiting, occur in 44%-57% of cases, while dyspepsia and weight loss are reported in approximately 43% of patients [10,11].

Neoadjuvant chemotherapy

Neoadjuvant chemotherapy is administered in approximately 10%-57% of patients before PD [11,13,16].

Preoperative imaging and assessment of pancreatic invasion

Preoperative imaging modalities, including computed tomography (CT) and endoscopic ultrasound (EUS), are routinely used for staging gastric cancer and may aid in the identification of pathologic tumor invasion. However, the diagnostic accuracy of CT and EUS for assessing the depth of pathologic tumor invasion has been reported to range from 77.1% to 88.9% and from 65% to 92.1%, respectively [17,18].

As a result, definitive confirmation of pancreatic invasion is ultimately established by histopathologic examination following surgical resection [9]. Nevertheless, intraoperative assessment of direct pancreatic tumor invasion remains challenging, with reported diagnostic accuracy rates ranging from 39% to 56.7% [17].

Symeonidis et al. reported that up to 55% of cases treated intraoperatively as direct pancreatic invasion were ultimately attributed to inflammatory reactions on final pathology [18]. Similarly, Piso et al. found that intraoperative suspicion of pancreatic tumor infiltration was histologically confirmed in only 39% of cases [13]. In contrast, Makuuchi et al. reported a higher confirmation rate of 72.7% when pancreatic invasion was suspected intraoperatively [17].

Surgical procedure

In patients with primary gastric cancer, all individuals underwent distal gastrectomy, whereas those with carcinoma arising in the gastric remnant underwent total gastrectomy [10]. In the overall population of patients with gastric cancer undergoing PD, total gastrectomy is performed in 64.4% of cases, while subtotal gastrectomy is performed in 35.6% [9].

Intraoperative blood loss during PD has been reported to range from 600 to 1,600 mL (overall range: 450-1,600 mL), with a mean operative time of approximately eight hours [10,11].

Reported median postoperative hospital stay ranged from 15 to 37 days, with overall ranges between 25 and 92 days [10-12,16].

Pathologic findings

The mean tumor size has been reported to range from 4 to 9 cm, with an overall range of 2.8-9.5 cm [4,6,9,12,16]. Histopathologic examination of surgical specimens revealed adenocarcinoma in all cases [10,11]. Well-differentiated tumors accounted for 23% of cases, whereas 74%-83% were classified as moderately or poorly differentiated [9,16]. Lymphovascular invasion has been reported in 85%-91% of patients [12,16], and perineural invasion was present in 70.8% of cases [16]. No patients were classified as N0 disease (0%) [16].

Resection margins

R0 resection was achieved in 71% to 87.5% of cases across reported series [9,11,12].

Postoperative morbidity and mortality

Reported postoperative morbidity rates range from 59.4% to 73.9% [10,11,19], with postoperative pancreatic fistula being the most frequent complication (31.2%-43.5%). Postoperative mortality has been reported at 4.2% [16].

Adjuvant therapy

Adjuvant therapy was administered in 45%-57% of patients [16,19]. Notably, Chan et al. reported the use of adjuvant chemotherapy in 100% of cases [11].

Recurrence patterns

Tumor recurrence has been reported in 56%-75% of patients, occurring between five and 48 months after PD [4,9,10,12]. According to Wang et al., the most frequent sites of recurrence were the gastric and pancreatic bed (35%), followed by the liver (18%) and retroperitoneal lymph nodes (12%) [9]. Similarly, Saka et al. reported nodal recurrence in 65% of cases, with additional sites including the liver (35%), peritoneum (6%), lung (6%), and spleen (6%) [10].

Overall survival and follow-up

Median follow-up ranged from 13 months (range: 4-182 months) [10] to 17 months (range: 1-210 months) [19], during which 56.5% of patients died [19]. Median overall survival varied across studies, ranging from 13 months to 26 months [11,19,20].

Reported overall survival rates at one year ranged from 75.3% to 77% [5,14]. Two-year overall survival rates varied between 43% and 60% [11,12], while three-year overall survival rates ranged from 34% to 41.9% [9,19]. Five-year overall survival rates were heterogeneous, ranging from 23% to 39.3% across published series [10,12,19].

To operate or not to operate

Overall survival among patients with pancreatic involvement was significantly lower than that of patients without pancreatic invasion, with reported two-year and five-year overall survival rates of 42.6% and 23.3%, respectively, compared with 57.5% and 42.1% in patients without pancreatic invasion (p = 0.002) [12]. Similarly, Saka et al. reported that the overall five-year survival of patients with suspected tumor invasion of the pancreatic head was only 13.6%, regardless of whether PD was performed [10].

Makuuchi et al. further demonstrated that, among patients with suspected pancreatic invasion, five-year survival tended to be lower in those with pathologically confirmed pancreatic invasion compared with those without confirmed invasion (12.5% vs. 66.7%), although this difference did not reach statistical significance (p = 0.150) [17].

When a tumor or metastatic lymphadenopathy directly invades the pancreatic head or infiltrates the duodenum, PD appears to be the only surgical option capable of achieving an R0 resection [17]. However, Saka et al. reported that among patients who achieved R0 resection, there was no significant difference in survival between those who underwent PD and those who did not [10]. These findings suggest that negative resection margins, rather than the extent of surgery itself, represent a key favorable prognostic factor after PD (p = 0.0174) [9].

Taken together, reported R0 resection rates in selected cases of gastric cancer with suspected pancreatic involvement further support consideration of extended resection with curative intent when surgery is performed in high-volume centers [9,10,17].

Poor prognostic factors

Positive peritoneal cytology has been identified as a negative prognostic factor, with a reported hazard ratio of 3.470 (95% confidence interval (CI): 1.011-11.909; p = 0.048) [19].

Lymph node status represents a critical prognostic determinant in gastric cancer with pancreatic involvement following resection. Advanced nodal stages (N2 and N3) have been associated with significantly worse survival compared with N0 and N1 disease (p < 0.001) [12]. Nunobe et al. further demonstrated the prognostic impact of nodal burden by comparing patients with ≥7 versus ≤6 metastatic lymph nodes. Five-year survival rates were significantly higher in the ≤6 group than in the ≥7 group (p = 0.014) [21]. Among patients who achieved R0 resection, five-year survival reached 50.0% in the ≤6 group, whereas it was only 7.7% in the ≥7 group [21].

Patients presenting with adverse prognostic factors, including lymph node metastases, positive peritoneal lavage cytology, or peritoneal dissemination, exhibited a five-year survival rate of 0%, compared with 47.4% in patients without these factors [10].

Neoadjuvant chemotherapy appeared to confer a survival benefit, with improved outcomes observed among treated patients (p = 0.039) [7,11].

In a multivariate analysis, Jin et al. identified neoadjuvant chemotherapy (p = 0.020), tumor type (linitis plastica vs. non-linitis plastica; p = 0.033), nodal stage (p = 0.011), resection margin status (R0 vs. R1; p = 0.010), and postoperative treatment (p = 0.017) as independent prognostic factors. Among these variables, resection margin status (R0 vs. R1) emerged as the strongest prognostic determinant [16].

Nonoperative (palliative) management

Among patients who did not undergo PD, the reported one-year and three-year overall survival rates were 41.7% and 5.6%, respectively [9]. Overall survival in the palliative group was significantly lower than that observed in patients who underwent PD (p = 0.0064) [9].

Hirose et al. further demonstrated that, among patients with gastric cancer invading the pancreatic head, median survival was significantly longer in those who underwent PD compared with those treated with palliative gastrectomy (19 months vs. 9 months; p = 0.0478) [22].

Taken together, these findings suggest a potential survival advantage associated with PD in carefully selected patients with distal gastric carcinoma invading the pancreas [22,23].

Meaningful statistical comparisons are limited by the rarity of PD in gastric cancer and the highly selected nature of these patients; therefore, available evidence is largely derived from small retrospective series. In this context, when preoperative imaging suggests that a Whipple procedure may be required to achieve oncologic clearance, multidisciplinary evaluation and thorough informed consent within experienced tertiary care centers are essential [3].

From a technical standpoint, PD performed for gastric cancer invading the pancreas is essentially identical to the Whipple procedure performed for primary pancreatic malignancies. However, available series suggest that patients undergoing PD for gastric cancer tend to experience higher overall morbidity, longer hospital stays, and slightly higher perioperative mortality compared with those operated on for primary pancreatic malignancies, even when surgery is performed in high-volume centers. In contrast, long-term oncologic outcomes appear heterogeneous in both settings and are largely influenced by differences in tumor biology, disease extent, and patient selection rather than surgical technique alone. These considerations underscore that indications for PD in gastric cancer should be driven primarily by the feasibility of achieving an R0 resection and appropriate oncologic selection, rather than technical considerations alone [24].

Limitations

This review has several limitations that should be acknowledged. First, the available literature on PD for gastric cancer with pancreatic involvement is limited and consists predominantly of small retrospective series, reflecting the rarity of this clinical scenario. Consequently, heterogeneity exists across studies in terms of patient selection, staging criteria, surgical indications, and perioperative management.

Second, given the narrative and non-systematic nature of this review, no formal quantitative synthesis or risk-of-bias assessment was performed. Therefore, the findings should be interpreted as descriptive rather than definitive.

Finally, comparisons between patients undergoing PD and those managed nonoperatively or with less extensive surgery are subject to inherent selection bias, as candidates for PD are highly selected based on disease extent and performance status. Despite these limitations, the consistency of findings across multiple series supports the central conclusion that achievement of R0 resection, rather than the extent of surgery itself, is the primary determinant of survival in this setting.

## Conclusions

PD is not routinely indicated for gastric cancer but may be justified in carefully selected patients with pancreatic involvement when required to achieve R0 resection. Available evidence suggests that long-term survival in this setting is primarily determined by negative resection margins and tumor biology, rather than by the extent of surgery itself.

High postoperative morbidity and the technical complexity of PD underscore the importance of strict patient selection, thorough preoperative evaluation, and performance in specialized centers. Advanced nodal disease, positive peritoneal cytology, and R1 resection are consistently associated with poor outcomes and should be considered contraindications to aggressive surgical approaches. In contrast, selected patients without adverse prognostic factors may derive a survival benefit from PD compared with nonoperative or palliative management. Future studies with larger cohorts and standardized selection criteria are needed to better define the role of PD in gastric cancer with pancreatic involvement.
